# The Egg Hub Model: A Sustainable and Replicable Approach to Address Food Security and Improve Livelihoods

**DOI:** 10.1016/j.cdnut.2024.103795

**Published:** 2024-06-13

**Authors:** Srujith Lingala, Mathilda Freymond, Puja Peyden Tshering, Priyanka Kumari, Klaus Kraemer, Kalpana Beesabathuni

**Affiliations:** 1 Sight and Life, Basel, Switzerland; 2 Department of International Health, Johns Hopkins Bloomberg School of Public Health, Baltimore, MD, United States

**Keywords:** eggs, rural, developing countries, smallholders, backyard poultry, agriculture, business models, scalability, food systems, low- and middle-income countries

## Abstract

**Background:**

Despite progress in improving living standards and reduced poverty, food insecurity and malnutrition remain a significant issue worldwide. Childhood is a critical time for the intake of protein to support physical and cognitive growth, including animal-source foods like eggs which can effectively mitigate stunting in low- and middle-income countries. In Malawi, high malnutrition rates among women and children represent a significant public health challenge, but high-quality sources such as eggs remain costly, scarce, and rarely consumed by children in Malawi. The Egg Hub model, identified and piloted by local agri-food entrepreneurs and the *Sight and Life* Foundation in Malawi, tackles the obstacles faced by smallholder farmers, working to increase egg production, enhance availability, and improve consumption within rural communities by supporting small-scale farmers transition from unsustainable and unproductive backyard rearing to small-scale farm through access to high-quality inputs, training, loans, and a guaranteed market for their eggs.

**Objectives:**

This paper provides a detailed account of the implementation of the pilot of the Egg Hub Model in Malawi including the demand creation process, the impact of the model on producers, consumers, and operators, and the social, economic, and environmental sustainability aspects of the model.

**Methods:**

Qualitative and quantitative surveys (n = 217 consumers) were used for demand creation and qualitative surveys were used with 15 retailers to determine egg sales. With 16 farmers and the egg hub operator, business metrics, including profits and loss records, were analyzed.

**Results:**

The pilot of the Egg Hub model in Malawi supported 85 farmers to triple their egg production, allowing their communities to purchase eggs at prices reduced by 40%, benefiting an estimated number of 180,000 rural poor. Egg consumption among the target population increased from an average of 2 to 9 eggs/month and led to reduced egg wastage and better biosecurity, reducing the risk of children’s exposure to chicken feces and infections. The achievements of this Egg Hub in Malawi allowed the model to be replicated in Ethiopia, Peru, and Brazil, producing 40 million eggs annually and benefiting more than half a million consumers.

**Conclusion:**

The Egg Hub model is a comprehensive and scalable solution to increase egg supply, address malnutrition and food insecurity, and improve livelihoods. The advantages include centralizing key activities through a community-centered approach, empowering female farmers, increasing access to a highly nutritious food, and economic benefits for farmers and their communities.

## Introduction

Considerable progress has been made on a global scale in improving living standards, reducing poverty, and lowering child and maternal mortality rates. Nevertheless, food insecurity and malnutrition persist, with numerous countries falling behind in achieving Sustainable Development Goal 2 aimed at tackling malnutrition by 2030. According to the latest findings from the State of Food Insecurity (SOFI) report 2023, ∼30% of the world’s population was moderately or severely food insecure in 2022, marking an increase of 13.5% compared with 2019 [1]. This upward trajectory in food insecurity might in part be attributed to the numerous challenges the world faced in 2022 – such as the postpandemic economic recovery, the conflict in Ukraine, and the soaring prices of food, agricultural inputs, and energy [1]. Furthermore, a substantial 22.3% of children under 5 were affected by stunting in 2022, with rates of 32.4% in Sub-Saharan Africa and 31.8% in South Asia [1]. The causes for stunting are multifaceted, yet poor complementary feeding practices and low-quality diets lacking essential vitamins, minerals, proteins, and other nutrients are key contributors to this burden [2]. The repercussions of childhood stunting are far-reaching, with long-term consequences on human capital. These include, increased morbidity and mortality, compromised cognitive development, diminished educational achievement, decreased future earning potential, and reduced overall productivity [3]. Across 95 low- and middle-income countries (LMICs), childhood stunting costs the private sector ≥USD 135.4 billion in sales annually [4]. Investing in stunting reduction interventions yields gains from USD 2 to USD 8 per USD 1 invested annually [4].

Research findings indicate that including animal-source foods in a child’s diet can effectively mitigate stunting in LMICs [5,6]. Moreover, regular egg consumption has been shown to significantly enhance child growth and cognitive development, while also improving dietary diversity and ensuring they receive sufficient nutrients to meet their dietary needs [[7], [8], [9], [10], [11]].

Eggs are known for being relatively affordable and particularly nutrient-dense, packing a significant amount of essential nutrients into every serving. They are an excellent source of protein and micronutrients; they contain all essential amino acids, 13 essential vitamins and minerals, docosahexaenoic acid, and choline – crucial components for growth, fetal brain development, and functioning [12]. In addition, eggs are easy to prepare and versatile, offering an outstanding opportunity to elevate and diversify the diets of both children and mothers.

Despite their apparent benefits, eggs remain costly, scarce, and rarely consumed by children in Sub-Saharan Africa and South Asia [13]. According to the Food and Agricultural Organization, in 2021, egg availability in Sub-Saharan Africa and South Asia was 40 and 87 eggs per capita per year, respectively, compared with 322 eggs in North America [14]. On the consumption side, data from the global UNICEF databases reveals that 22% of children between 6 and 23 mo globally consume eggs, with only 17% in the poorest households compared with 30% in the wealthiest households [15]. Although poultry husbandry plays an important role in the livelihoods of rural communities within developing countries [16], the existing poultry farming systems in these regions predominantly involve extensive backyard setups – systems that are unproductive, unsustainable, and even pose some health hazards [[17], [18], [19], [20]]. Emerging evidence suggests that greater exposure to *Campylobacter*-infected poultry or their feces elevates the risk of developing environmental enteric dysfunction, a chronic inflammation of the intestine, resulting in poor absorption of food, macro- and micronutrients [[15], [19], [21], [22]]. In addition, this condition is strongly associated with childhood stunting [23].

In light of the abovementioned challenges, continuing poultry rearing as usual will not improve egg availability and consumption in LMICs. An imperative emerges for the establishment of more productive, sustainable, and safer ways of both producing and accessing eggs in LMICs.

## The Innovation

### Identification of a scalable business model

In 2016, Beesabathuni et al. [17] investigated constraints in egg production and conducted a value chain assessment in 4 LMICs; Kenya, Ethiopia, Malawi, and India. These countries were selected after a comprehensive literature review and discussion with experts in the poultry sector, donors, and impact investors. After 160 key informant interviews with farmers, inputs suppliers, integrators, women self-help groups, and poultry social enterprises, Beesabathuni et al. [17] identified several challenges faced by smallholder poultry farmers, such as high inputs costs and limited access to chicks, feed, vaccines, extension services, markets, and credit. From the egg value chain assessment in 3 countries in East Africa and India, the authors profiled 5 business models; microfranchizing, microfinancing, co-operative farming, enterprise development, and out-grower model, all advanced by diverse actors: private companies, nongovernmental organizations, and microfinance institutions [17]. An analysis of these business models was conducted based on 4 criteria: impact, relevance, sustainability, and scalability. Although all 5 models involved smallholder farmers to increase egg production through access to soft loans, improved inputs, and extension services to varying degrees, 4 of these models additionally invested in the aggregation of smallholders to become more economically savvy trading partners and capitalize on economies of scale [17]. Beesabathuni et al. [17] found that the models were improving hen productivity from an average of 40 eggs per bird in the backyard setting to a minimum of 100 eggs per bird in the microfranchizing model and a maximum of 290 eggs per bird in the enterprise development and out-grower model. Smallholder farmers in the enterprise development and out-grower model are profitable, and have high annual incomes, ranging from 2 to 15 times more than the farmers in the other models. Comparatively, other models that aimed to improve backyard chicken-rearing practices with smaller flock sizes have not been profitable, with a modest impact on the household’s well-being and egg consumption [24]. Additionally, the capital and program costs per egg are the lowest, the benefits of which, when passed to the consumer, make eggs more affordable compared with traditional models. Overall, these country case studies validate the hypothesis that productivity and viability increase with farm sizes of 1000–5000 birds [17]. The out-grower and enterprise development models demonstrated significant potential for rapidly increasing egg yields. These models can achieve self-sufficiency, operate at or close to full capacity, and provide a high income for the farmers, as well as increase egg availability and affordability [17]. Following this analysis, the concept of the “Egg Hub” model was introduced by local agrifood entrepreneurs and *Sight and Life*, a nonprofit foundation, and qualified as a social business model designed to address the key bottlenecks that prevent adequate egg production and availability in low resource rural and peri-urban settings.

## Implementation Of The Egg Hub Pilot In Malawi

### The Malawian context

Nutritious diets remain largely unavailable to most Malawians, and even where they might be available, they are often unaffordable [25]. In 2020, the minimum dietary diversity indicator, defined as the consumption of ≥5 food groups out of the 8 referenced during the previous day, was 17% among children 6-23 months [26]. More than one-third of children under 5 y (34%) are stunted in Malawi, and iron deficiency anemia is a public health concern for children under 5 y and women of reproductive age with rates of 63% and 33%, respectively [27]. Additionally, egg availability in the country is very low, with an annual per capita average of 28 eggs [14]. This can be explained by the current poultry production models in Malawi primarily consisting of backyard operations. These models account for a staggering 85% of the industry and are suffering from inefficiency and high costs [28].

### The Egg Hub pilot

Between 2018 and 2021, with financial support from *Stichting Dioraphte* and in collaboration with a local implementing partner, *Sight and Life* piloted the very first Egg Hub in the rural and remote areas of Lilongwe, Malawi. The Egg Hub model was defined as a centralized unit providing high-quality affordable inputs, extension services, training, and market access to independent farmers involved in layer farming. The Egg Hub model organizes smallholder farmers into groups of 5 and provides an input package, training, and market support to sell eggs. Farmer groups are encouraged to buy improved feed at wholesale rates. Farmers sell eggs primarily in their communities. The trucks that deliver feed bring any excess eggs back to be sold in urban markets. Farmers pay back loans in 3–5 y which goes into a revolving fund that allows the hub to increase the number of farmers. The model creates an enabling ecosystem through improved infrastructure, feed supply, veterinary services, entrepreneurial training, farm management, and access to the market.

Currently, the Egg Hub in Malawi is operating at 85 farmers producing 8.6 million eggs each year. The consumer groups include convent nuns, all-women groups, schools, and urban poor living in slums.

## The Egg Hub Operational Model

### The Egg Hub operator as the first pillar of the model

The Egg Hub operator plays the role of an aggregator, responsible for providing smallholder farmers with necessary inputs such as feed, vaccines, medicines, training, and point-of-lay birds. The careful selection of an Egg Hub operator is critical to the success of the model. A feed miller is best positioned to play the role of an Egg Hub operator because 70% of the total cost attributed to an egg stem from feed expenses [29]. Additionally, the efficacy of an optimally formulated feed impacts the entire production chain. It has a profound influence on bird productivity and farmer profitability [29,30]. The Egg Hub operator addresses the challenges of an independent poultry farmer by *1*) sourcing raw materials for feed in bulk and transferring the benefits of economies of scale to the farmers in the form of affordable feed, *2*) delivering feed to them at their doorstep, *3*) customizing feed recipe that aligns with different hen requirements, such as calcium-enriched feed for aging hens, *4*) supplying them with vaccinated and well-raised pullets that are ready to lay eggs, thus enabling them to generate revenue through the sale of eggs immediately and not having to wait 16 wk for day-old chicks to turn pullets, and *5*) mitigating biosecurity threats by providing an on-call veterinarian, always with the goal of helping the farms thrive.

### Small-scale poultry farmers selection, benefits, and demographics

Poultry farmers were organized into groups of 5 per farm and they were selected from nearby villages, based on specific criteria: *1*) the chosen villages were situated within a 100-km radius of the Egg Hub, *2*) they had consistent access to a national highway or well-maintained roads for convenient feed transportation, and *3*) they were located <5 km away from a trading center or open market to facilitate the sale of eggs. To qualify for selection, poultry farmers had to meet the 4 following conditions: *1*) they were required to own land and construct appropriate sheds for their farms, *2*) among the group of 5 farmers, ≥1 farmer had to live close to the farm to facilitate ongoing monitoring of the farm’s performance, *3*) 2 or 3 farmers within the group were expected to have prior experience in livestock farming, and *4*) the farmer group would have to operate without hiring external labor for farm management, undertaking all tasks themselves.

By joining the Egg Hub, farmers benefit from access to better quality inputs at stable prices, such as feed delivered consistently at fixed rates, shielding them from immediate market fluctuations. Additionally, instead of purchasing day-old chicks, farmers would have the opportunity to buy vaccinated and well-raised 18-wk-old pullets that are ready to lay eggs [14]. Consequently, the high mortality risk associated with rearing birds until 18 wk is borne by the operator and not the farmers. The operator is able to mitigate the risk by adapting essential measures such as the provision of appropriate infrastructure, stringent biosecurity measures, and the application of effective poultry rearing practices. Farmers, having received 18-wk-old pullets, begin to earn revenue immediately. The Egg Hub operator would also provide training and on-farm support, equipping farmers with the knowledge and skills needed for successful poultry farming. Furthermore, the Egg Hub would offer extension services, continuously monitoring farm productivity and assistance in case of issues related to bird health or diseases. This streamlined approach to farming would simplify the process for farmers, as all necessary inputs are delivered to their doorstep. Another significant advantage would be the absence of working capital costs, as the Egg Hub would cover upfront expenses such as feed, vaccines, and medicines. Moreover, farmers would have the safety net of selling their eggs back to the hub if they encounter difficulties selling within their community, providing added security and support. The Egg Hub creates self-reinforcing feedback loops that continuously strengthen the business model, a powerful, yet neglected aspect of creating a business model [31]. The operator provides high-quality feed, birds, and vaccines to the farmers. The farmers, in turn, ensure proper care, housing, and management for the birds, resulting in increased egg production, faster growth, and better quality meat, contributing to higher income for farmers. Growing demand for high-quality inputs from farmers results in increased sales and reputation for the operator. A feedback loop is established as farmers consistently patronize the Egg Hub, leading to mutual success. This virtuous cycle locks in quality, as farmers’ success relies on the consistent provision of inputs by the Egg Hub, and the Egg Hub’s prosperity depends on the farmers’ commitment.

An internal baseline survey conducted among 16 early affiliate farmers, the first group of farmers who signed up to join the Egg Hub, showed that this group typically fell within the middle-income bracket, owning assets such as land, houses, some livestock, bicycles, and occasionally motorcycles (Table 1). ∼60% reported daily earnings exceeding USD 2.5. Additionally, 75% of these farmers displayed a strong entrepreneurial mindset, as evidenced by their history of taking loans from lending agencies to kickstart various businesses [32]. All early adopters came equipped with prior experience in livestock farming, primarily engaging in backyard rearing of animals such as goats, pigs, and indigenous chickens. In the context of the Egg Hub, the primary financial risk they faced was the construction of poultry houses. Although the investment for this construction was substantial, its shared nature among group members mitigated the potential for overwhelming debt burdens, making it a more manageable risk for these forward-thinking farmers.

**TABLE 1:** Descriptive statistics of the baseline survey among Egg Hub farmers (*n* = 16)

Variable		*n* = 16	Percentage (%)
Sex of the farmer	Female Male	5 11	31.25 68.75
Age of the farmer (y)	25 or less 26–30 31–35 36–40 41–45 46–50 51–60	1 2 3 5 2 1 2	6.25 12.50 18.75 31.25 12.50 6.25 12.50
Price of eggs in the nearest market (in Malawian Kwacha)	75 MWK 90 MWK 100 MWK	7 3 6	43.75 18.75 37.5
Productive assets owned in the household (>1 answer possible)	Motorcycle Bicycle, land, livestock, and house	7 16	43.75 100.00
Household earning/d (in USD)	<$2.5/d $2.5–$5/d >$5/d	6 5 5	37.5 31.25 31.25
Current source of monthly income (>1 answer possible)	Piece work Private personal business Farming	2 3 16	12.50 18.75 100.00
Eggs consumed by your family last week	10 or less 11–20 31–40	10 2 4	62.50 12.50 25.00
Whether loan was taken last year	Yes No	12 4	75.00 25.00
Loan was taken from	Microfinance support Church/Community Based Organization/Non-Governmental Organization Friends/family Village Savings/Loan Association Tobacco/other farming entity No loan taken	1 1 1 3 6 4	6.25 6.25 6.25 18.75 37.50 25.00
Whether they were in any livestock farming previously	Yes	16	100
Animals they reared (>1 answer possible)	Cattle Goats Local chickens Pigs Other animals	5 12 13 15 6	31.25 75.00 81.25 93.75 37.50
Whether they were involved in layer poultry farming before the Egg Hub intervention	No	16	100.00

### The Egg Hub’s loan replacement mechanism

In the framework of the Egg Hub model, poultry farmers were relieved from any of the upfront costs for raising the day-old chicks to pullets, vaccines, and medicines – costs that are typically shouldered by independent farmers. These financial burdens were borne by the Egg Hub. Furthermore, the Egg Hub’s loan replacement mechanism provided smallholder farmers with access to zero-interest loans, a significant development in many low- and middle-income regions where traditional loans are inaccessible as they are not able to provide collateral or because of high commercial interest rates [33]. Through this mechanism, the Egg Hub loaned out the first batch of pullets to farmers. Subsequently, as farmers generated income from egg sales, they used these earnings to purchase the pullets. Feed costs and a portion of the loan were collected from farmers on a weekly basis.

Because the Egg Hub’s role involved providing input as credit rather than direct monetary transfers, it operated outside the scope of certain legal requirements. Nonetheless, to uphold transparency between farmers and the Egg Hub operator, both parties entered a contract that outlines the terms of credit, solidifying their commitment. In situations where a farmer encountered challenges in repaying the loan on time, the operator closely monitored the circumstances and took appropriate decisions to solve the issue. Usually, a grace period of 1 mo was granted to farmers to settle their debt before any actions were considered.

### The demand creation process

For a well-functioning Egg Hub model to be truly effective, it was essential not only to increase egg production and availability but also to ensure these eggs were consumed by the target populations, including pregnant and lactating women and mothers with ≥1 child under 5 y of age. To fulfill this objective, *Sight and Life* developed a comprehensive social marketing campaign. This campaign was crafted based on insights derived from formative research, encompassing existing knowledge, relevant attitudes, behaviors, cultural nuances, as well as religious, social, and familial influences around egg consumption.

Field-based formative research involves the analysis of knowledge, attitudes, perceptions, current consumption patterns, and barriers to the consumption of eggs. The research was conducted from a selective sampling of our target beneficiaries, pregnant women, lactating mothers, and children under 5 y. The data was gathered through open-ended interviews and focus group discussions. The formative research showed that Malawian mothers were willing to do more and find newer ways to keep their families healthy and happy. The Campaign’s Big idea was then developed and coined as: “Grab every opportunity to make your family healthy and happy!” This message encapsulated the essence of the campaign’s mission.

To represent the Malawi Egg Hub eggs, the brand name “Zonse Momo” was chosen. This term means “all-encompassing” or “selfcontained” and was open to interpretation in many ways, whether from the perspective of the consumers or the poultry farmers.

The targeted social marketing campaign was launched as a mix of mass media, interpersonal, and point-of-sale interventions for 6 mo.

Finally, to evaluate the campaign effectiveness, data, such as interviews and surveys, was collected from 415 consumers at 3 time-points, before the social marketing was launched (baseline), at the mid-point (after 3 mo; midline), and at the campaign end (after 6 mo; endline). Findings will be shown in the value chain impact, consumers section below.

## Value Chain Impact of the Egg Hub in Malawi

This section explores the impact of Egg Hub on the broader egg value chain and its implications for various stakeholders in Malawi.

### Impact on the Egg Hub farmers

Through effective farm management practices, consistent access to quality inputs, improved breeds, and the implementation of biosecurity measures, hens’ egg-laying capacity surged from ∼30–80 eggs/y in backyard farming to 292 eggs/y postimplementation of the Egg Hub [17,34]. The Egg Hub farmers reported a lower mortality rate adjusted for flock size, 3%, compared with the predominant backyard model with 60% mortality rates [16,35]. In total, three-and-a-half million fresh eggs were produced every year. Egg Hub eggs, twice the weight of backyard poultry eggs, had thicker shells, resulting in significantly reduced losses during transportation and storage [34]. This increase made Egg Hub eggs more affordable at a third of the price of the backyard egg, on a unit weight basis [34].

**Figure 1:**
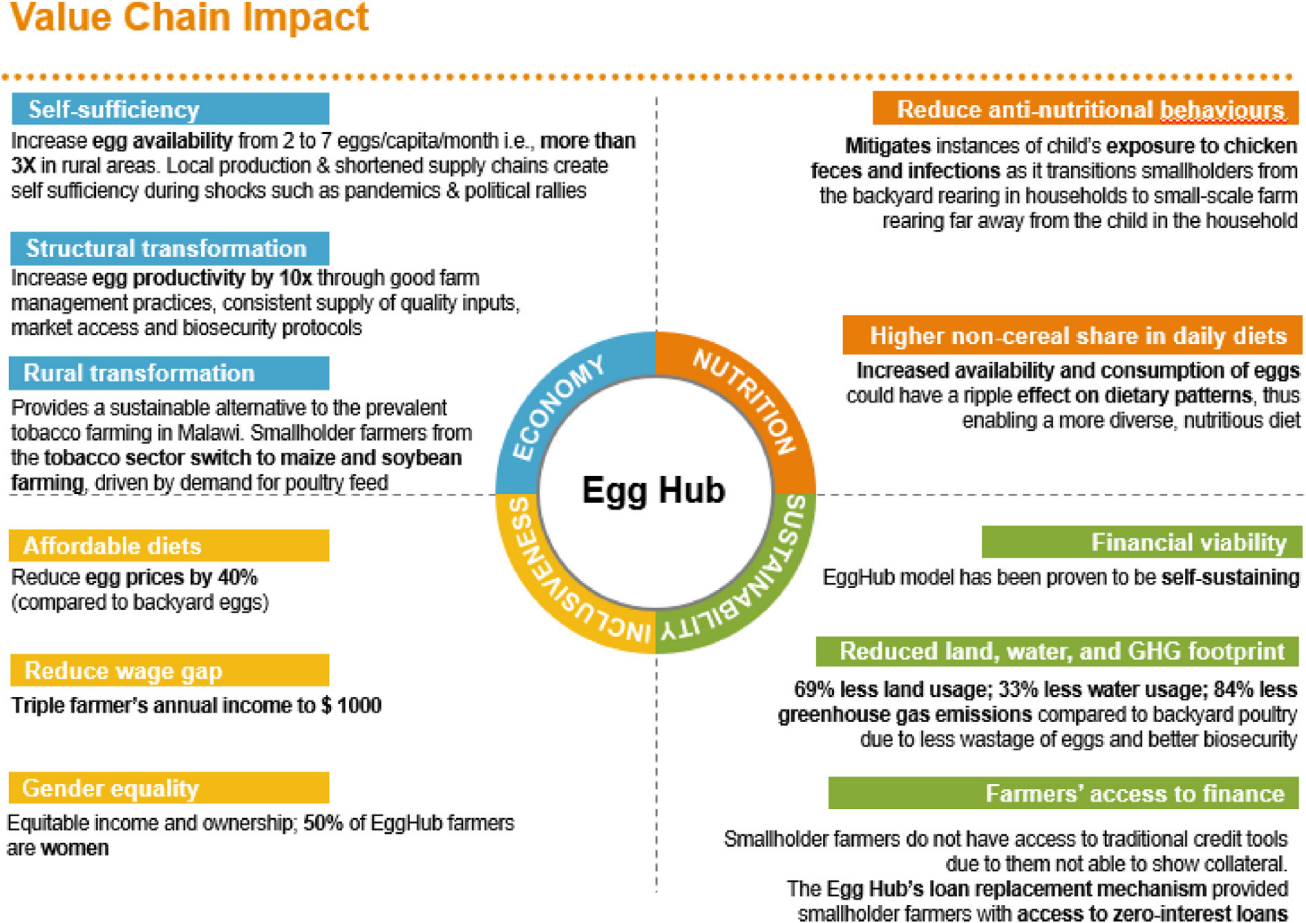
Value Chain Impact of the Egg Hub Model in Malawi. *Source: Sight and Life team analysis based on IFAD’s Magnet Food System Indictors*

Although the eggs were primarily sold within the farmers’ local communities, any surplus eggs were channeled to retail centers, including urban markets, further expanding their reach. The annual income of Egg Hub-affiliated farmers increased from USD 300 to USD 1000 (Figure 1) [34]. Furthermore, on the market, 75% of surveyed shopkeepers acknowledged sourcing eggs from Egg Hub-affiliated farms. However, 87.5% of these shopkeepers continued to procure eggs from other providers as well, indicating that the Egg Hub did not eliminate other farms. This aligns with the overarching project goal of increasing the overall egg supply [35].

These numerous benefits, along with the extension services and improved farming conditions, contributed to a reported 95% repayment rate among farmers [34].

### Impact on the consumers

The Malawi Egg Hub reached ∼210,000 individuals in rural poor communities [34]. It also enhanced egg availability, with the monthly egg supply per person surging from 2 to 7, a 3-fold increase in rural areas (Figure 1) [34]. Throughout this program, egg prices were reduced by 40% in comparison to backyard eggs, reaching USD 0.10/egg [34].

Furthermore, the social marketing campaign had a positive impact on awareness, egg purchasing, and consumption within the target demographic of mothers and young children [36]. The data was collected at 3 time-points; at baseline, at midline (after 3 mo), and at the project end (after 6 mo). The same people were interviewed at each time point. Overall, the social marketing campaign and interventions increased the consumption of eggs. Over half of the target audience (51%, *n* = 217) ate eggs twice or more a week at the project’s end. This was compared with only 12% (*n* = 229) at baseline. Only 7% did not eat eggs at all at the endline, compared with 55% at baseline [36]. Furthermore, increased availability and consumption of eggs could have a ripple effect on dietary patterns.

Lastly, a possible added benefit of the Egg Hub could be fewer cases of children coming into contact with chicken feces and getting ill. This could happen because the model shifts smallholders away from household backyard rearing to small-scale farm rearing.

### Impact on the Egg Hub operator

Managing the operations of the Egg Hub proved to be financially rewarding for the operator. Beyond this immediate gain, the Egg Hub offered a lucrative growth strategy. The operator had the opportunity to cultivate partnerships with farms, positioning himself as the primary supplier during the initial stages and scaling alongside the farms as their poultry flocks grow. This approach enabled the Egg Hub operator to nurture and maintain farms as long-term clients, effectively establishing the Egg Hub model as a valuable pipeline for customer development [35]. The social marketing campaign also enabled the Egg Hub operator to create an appeal for farmers to join the Egg Hub as a lucrative means of livelihood.

## Social, Economic, and Environmental Sustainability

The Egg Hub’s ability to sustain itself was demonstrated in the first pilot. More than half of Egg Hub farmers are women, who, as the earning members of their families, are empowered to make financial decisions that positively impact themselves and their families (Figure 1) [34]. It also made the community self-sufficient by improving access to eggs locally. This effect was sustained through the COVID-19 pandemic and the community got access to a nutritious diet at affordable prices.

Although the first group of Egg Hub-affiliated farmers achieved a break-even point within 3 y, solidifying their status as established poultry farmers, the Egg Hub operator achieved break-even within 5 y, establishing a profitable and viable venture [35]. The loans collected from the farmers contributed to the establishment of a revolving fund. This fund, managed by the Egg Hub operator, supported the financing of a subsequent cohort of farmers over the following 3 y, thereby facilitating a well-managed expansion strategy that ensured continued growth and sustainability, with no dependence on external funding.

Furthermore, the Malawi Egg Hub implements strict protocols on farm management and biosecurity practices, such as constant monitoring of bird health. This resulted in decreased egg wastage, increased egg productivity, and improved bird health, impacting an estimated 69% reduced land demand, 33% lower water footprint, and 84% less greenhouse gas emissions than backyard poultry (Figure 1) [1,[37], [38], [39], [40]]. Additionally, the Egg Hub incorporated circular economy practices by repurposing chicken manure as fertilizer for crops and selling spent birds for meat.

## Discussion

The objective of the first initiative of the Egg Hub model in Malawi was to produce and create demand for high-quality, affordable, and nutritious food, aiming to combat malnutrition and poverty sustainably. By providing smallholder poultry farmers with high-quality affordable inputs, intensive training, zero-interest loans, and marketing support, the Egg Hub model increased productivity and reduced bird mortality and biosecurity risks, leading to significant improvements in egg availability in the region. Reducing inefficiencies associated with food production can lead to significant improvements in the value chain. Central to the success of the model was the pivotal role of the hub manager, who served as the primary link for supplying feed, medicines, vaccines, and birds and also served as a potential market channel for farmers’ produce – eggs.

The innovative model of the Egg Hub offered 3 specific benefits over traditional farming models. First, it centralized key activities, leveraging expertise to achieve scale, thereby ensuring efficient resource allocation and management. Second, the model not only ensured food and nutrition security and economic benefits but also localized these advantages to rural populations, fostering sustainable growth in these areas. Third, the Egg Hub model provided strong economic motivation to farmers, creating a pathway toward self-sufficiency and prosperity. Additionally, the hub benefits farmers from other agricultural sectors – maize and soy, among others – which are a source of feed for the hens. This resulted in smallholder farmers from the prevalent tobacco sector switching to maize and soybean farming, driven by increasing demand for poultry feed.

Furthermore, context-specific factors played a pivotal role in driving the achievements of this first implementation in Malawi. The Egg Hub significantly profited from its management as a thriving local business bringing essential scale and expertise, including established supplier relationships, expert knowledge in bird farming, and the inherent scalability. Moreover, the effective, targeted social marketing campaign increased brand awareness and egg consumption among mothers and children under 5 y.

The Egg Hub model, as highlighted in the 2023 SOFI report [1], presents a comprehensive and scalable win-win solution for small-scale producers and low-income consumers, offering a range of benefits. These advantages include providing access to one of nature’s most nutritious foods, empowering women in both domestic and agricultural contexts, improving animal and human health, reducing post-production losses, and decreasing dependence on grants. There are however limitations to this model. Despite its potential to enhance dietary diversity and food security, the Egg Hub currently lacks a comprehensive assessment of its actual impact on nutritional outcomes, such as stunting. To gain an understanding of its effectiveness, there is a need for an impact assessment study that evaluates how the Egg Hub contributes to improving nutrition within the communities it serves. One other notable limitation of the Egg Hub model is its reliance on a revolving fund as the primary financial mechanism for supporting farmers. This limitation becomes evident in the slow rate of farmer expansion, which doubles only every 3 y, hampering the rapid scalability of the program. In the Malawi context, it would take decades to expand the Egg Hub model throughout the country without additional funding. Consequently, it is imperative to secure additional funding to further scale and accelerate the positive impact of this model.

Nevertheless, building upon the solid foundation laid by this initial pilot, *Sight and Life* plans to reach a 1.5 million rural population by 2027. Moreover, the achievements of this first Egg Hub implementation have paved the way for the replication of the model in Ethiopia, Peru, and Brazil, producing a total of 40 million eggs annually and benefiting more than half a million consumers, thus establishing its scalability in different geographies and potential for transformative impact on a global scale.

## Author contributions

The authors’ responsibilities were as follows – KB, SL, KK: supported entrepreneurs—Andrew and Maya Stewart in the design and implementation of the Egg Hub model and subsequent scale-ups, PPT: implemented the social marketing campaign and consumer insights, MF: was responsible for writing and editing the manuscript, SL, PPT, PK: contributed content to the article, KK: critically reviewed and edited the manuscript, KB: led the submission process; all authors: contributed to the revisions contained in the article; and all authors: read and approved the final manuscript.

## Conflict of interest

The authors report no conflicts of interest.

## Funding

The Egg Hub implementation and scale-up were possible because of the support from Stichting Dioraphte.

## Data availability

Data described in the manuscript, code book, and analytic code will be made available upon request pending application and approval.
